# Hexb enzyme deficiency leads to lysosomal abnormalities in radial glia and microglia in zebrafish brain development

**DOI:** 10.1002/glia.23641

**Published:** 2019-05-29

**Authors:** Laura E. Kuil, Anna López Martí, Ana Carreras Mascaro, Jeroen C. van den Bosch, Paul van den Berg, Herma C. van der Linde, Kees Schoonderwoerd, George J. G. Ruijter, Tjakko J. van Ham

**Affiliations:** ^1^ Department of Clinical Genetics, Erasmus MC University Medical Center Rotterdam Rotterdam The Netherlands

**Keywords:** brain disease, glia, hexb deficiency, in vivo imaging, zebrafish

## Abstract

Sphingolipidoses are severe, mostly infantile lysosomal storage disorders (LSDs) caused by defective glycosphingolipid degradation. Two of these sphingolipidoses, Tay Sachs and Sandhoff diseases, are caused by β‐Hexosaminidase (HEXB) enzyme deficiency, resulting in ganglioside (GM2) accumulation and neuronal loss. The precise sequence of cellular events preceding, and leading to, neuropathology remains unclear, but likely involves inflammation and lysosomal accumulation of GM2 in multiple cell types. We aimed to determine the consequences of Hexb activity loss for different brain cell types using zebrafish. Hexb deficient zebrafish (*hexb^−/−^)* showed lysosomal abnormalities already early in development both in radial glia, which are the neuronal and glial progenitors, and in microglia. Additionally, at 5 days postfertilization, *hexb* ^−/−^ zebrafish showed reduced locomotor activity. Although specific oligosaccharides accumulate in the adult brain, *hexb^−/−^)* zebrafish are viable and apparently resistant to Hexb deficiency. In all, we identified cellular consequences of loss of Hexb enzyme activity during embryonic brain development, showing early effects on glia, which possibly underlie the behavioral aberrations. Hereby, we identified clues into the contribution of non‐neuronal lysosomal abnormalities in LSDs affecting the brain and provide a tool to further study what underlies the relative resistance to Hexb deficiency in vivo.

## 1 INTRODUCTION

Lysosomal storage disorders (LSDs) comprise a group of at least 55 disorders, which are caused by genetic mutations in lysosomal enzymes leading to loss of protein function (Kielian, 2019). This typically leads to storage of undigested material in lysosomes, and cellular dysfunction or cell death. LSDs frequently involve progressive neurodegeneration, and for most of them, there is no treatment available yet. How lysosomal dysfunction precisely leads to neurodegeneration in LSDs is not completely understood. One LSD, Sandhoff disease, is caused by mutations in *HEXB*, leading to deficiencies in β‐Hexosaminidase hydrolases, formed either by HEXA/B or HEXB/B dimers (Mahuran, 1991; Utsumi et al., 2002). Recessive mutations in *HEXA* cause Tay‐Sachs disease, which presents with identical clinical pathology to SD (Gravel, Triggs‐Raine, & Mahuran, 1991). β‐Hexosaminidase plays a role in ganglioside metabolism and hydrolyzes GM2 gangliosides into GM3 gangliosides in lysosomes, which in turn is further degraded by other lysosomal enzymes. Loss of either *HEXA* or *HEXB* results in accumulation of the ganglioside GM2, and therefore these disorders are clustered as GM2 gangliosidoses. GM2 accumulation is mainly found in brain cells, including neurons and glia (Kawashima, Tsuji, Okuda, Itoh, & Nakayama, 2009; Kyrkanides et al., 2012; Tsuji et al., 2005). It is unknown however, what molecular and cellular events precede pathology and cause SD or Tay‐Sachs disease.

In the most common and severe form of SD, symptoms appear during infancy. At about 3 to 6 months after birth, after an initially normal postnatal development, development slows down and skeletal muscles weaken, causing loss of acquired motor abilities such as turning over, sitting upright, and crawling (Hendriksz et al., 2004). Most symptoms are neurological and include an exaggerated startle response to loud noises, seizures, vision and hearing loss, intellectual disability, and paralysis. There is no treatment available and infantile Sandhoff patients typically do not live longer than 5 years (Hendriksz et al., 2004). Due to the very early onset and rapid disease progression, the initial cellular events leading to neuronal loss remain obscure.

SD neuropathology involves neuronal loss, the presence of increased numbers of activated microglia—the brain's macrophages— and astrocytes (Jeyakumar et al., 2003; Myerowitz et al., 2002; Sargeant et al., 2012). Expansion of both microglia and astrocyte numbers are in fact found in many brain disorders including epilepsy, other LSDs, multiple sclerosis, and neurodegenerative disorders, and likely play a role in pathogenesis (Fakhoury, 2018; Geloso et al., 2017; Joe et al., 2018; Lee et al., 2016; Oosterhof et al., 2018; Ponath, Park, & Pitt, 2018; Zhao et al., 2018). In SD mice, GM2 storage is found in lysosomes of neurons, but also in lysosomes of astrocytes and microglia (Kawashima et al., 2009; Kyrkanides et al., 2012; Tsuji et al., 2005). Based on pathology and gene expression data, it is still unclear which cell types are responsible for the initiation and progression of the pathology. In SD mice, microglial activation was found to precede neurodegeneration (Wada, Tifft, & Proia, 2000). In microglia, *HEXB* is remarkably highly expressed compared to expression in other brain cell types and even considered to be a microglial signature gene (Artegiani et al., 2017; Bennett et al., 2018; Butovsky et al., 2014; Gosselin et al., 2017; Hickman et al., 2013; Oosterhof et al., 2017). Given that microglia are highly phagocytic, lysosomal function is critical for processing ingested material and HEXB could also be important for microglial function. Several studies showed that suppression of microglial or astrocytic inflammation could reduce SD pathology, suggesting both glial cells might be involved in SD pathogenesis (Tsuji et al., 2005; Wada et al., 2000; Wu & Proia, 2004). It is important to determine if, and to what extent, HEXB deficiency also affects glial function or if gliosis is a consequence of neuronal problems directly caused by the loss of HEXB. Analysis of *hexb^−/−^* mouse fetal neuronal stem cells (NSCs), also called radial glia, revealed a more frequent differentiation towards the astrocyte lineage rather than neurons, indicating abnormal differentiation of brain cells (Ogawa et al., 2017). SD patient IPS cell‐derived organoids also showed aberrant neuronal differentiation (Allende et al., 2018). These studies suggest that *HEXB* deficiency could intrinsically affect neuronal differentiation and induce increased astrocyte numbers, which could be an initiating event in the pathogenesis of SD.

Potential therapeutic options for SD include gene therapy, immune suppression, and bone marrow transplantation. Gene therapy in SD mouse models, applied by intravenous viral transduction from postnatal day one and two, prevented pathology (Niemir et al., 2018). However, the subclinical disease process preceding the onset of symptoms makes early intervention using gene therapy difficult to implement. Immune suppression or bone marrow transplantations improve neurological function in SD mice, likely by reducing gliosis (Abo‐Ouf et al., 2013; Ogawa et al., 2017; Ogawa et al., 2018; Wada et al., 2000; Wu, Mizugishi, Bektas, Sandhoff, & Proia, 2008). Although these therapies seem very promising, it is important to understand to what extent such approaches are targeting the cause, rather than inhibiting the symptoms. Therefore, understanding the early cellular pathogenic processes will facilitate the development of an effective treatment for SD.

Remarkably, natural occurring *HEXB* or *HEXA* variants leading to gangliosidoses, have been observed in various domesticated as well as wild vertebrate species including dogs, cats, sheep, Muntjak deer, and flamingos (Cork et al., 1977; Kanae et al., 2007; Kolicheski et al., 2017; Martin et al., 2004; Rahman et al., 2012; Torres et al., 2010; Zeng et al., 2008). The disease course in these animals is similar to that in humans, showing the conserved importance of proper processing of gangliosides. To understand SD pathogenesis, we aimed to visualize the earliest lysosomal abnormalities to discern the affected cell types during brain development in vivo. We used the zebrafish as a model since their offspring develop ex utero allowing the visualization of pathology in vivo using live imaging, they have a relative small size and are transparent, and have previously been used to study lysosomal (dys)function (Berg et al., 2016; Festa et al., 2018; Keatinge et al., 2015; Li et al., 2017; Louwette et al., 2013; Shen, Sidik, & Talbot, 2016). Moreover, zebrafish harbor a single *HEXB* homolog, *hexb*, and transgenic reporter lines are available to label different cell types. We generated an SD zebrafish model in which we investigated the earliest emergence of cell‐type specific lysosomal abnormalities in the developing brain. Already in the first days of brain development, we identified increased lysosomal volume in radial glia and altered lysosomal morphology in microglia. With the present study, we identified some of the cellular consequences of loss of Hexb enzyme activity during embryonic brain development, showing early effects on glia, which possibly underlie the behavioral aberrations.

## 2 RESULTS

### 2.1 Direct mutagenesis causes Hexb enzyme deficiency and glial lysosomal abnormalities

**Figure 1:**
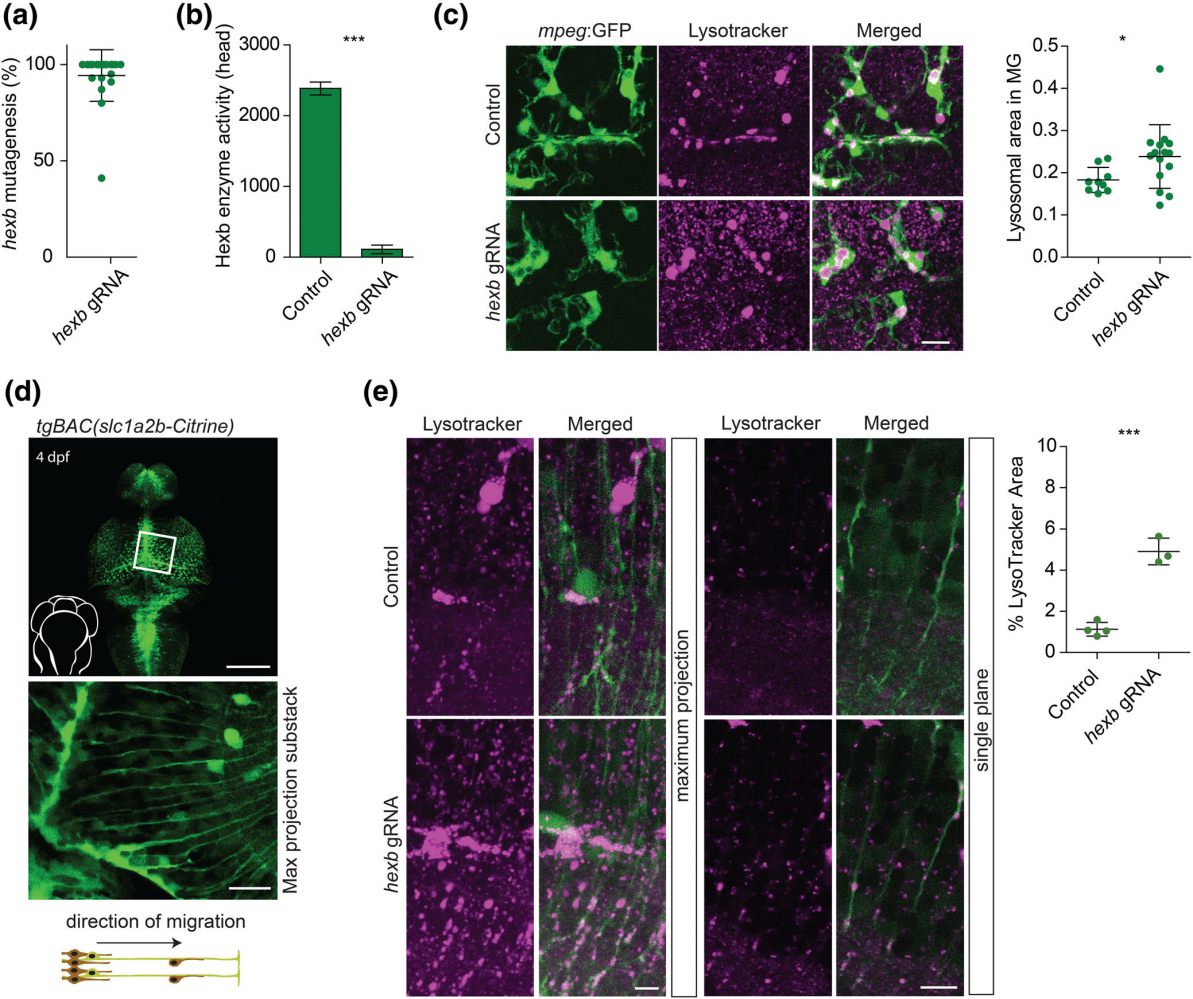
Efficient mutagenesis in *hexb* crispants shows abnormal lysosomes in microglia and radial glia. (a) graph shows mutagenesis efficiency for 24 injected fish at 4 dpf. Each dot represents 1 larva. (b) *hexb* crispants show almost no enzymatic conversion of substrate by β‐Hexosaminidase A + B at 4dpf. (c) Representative images of microglia in 8 dpf larvae imaged in the depicted area. Scale bar represents 20 μm. Quantification of lysotracker (LT+) area within microglia relative to the total microglial area in 8 dpf larvae. Each dot represents 1 larva. (d) Expression pattern of the tgBAC(*slc1a2b*:*Citrine*) transgenic line at 4 dpf, with magnification/sub‐stack showing the radial fibers in the optic tectum, and a schematic showing radial glia orientation and direction of neurogenic migration along the radial fibers. Scale bars represent 100 and 20 μm, respectively. (e) Representative images of radial glia at 4 dpf in controls and in *hexb* crispants with quantification of relative LT+ signal per measured total area. Each dot represents 1 larva. Scale bars represent 10 μm. Error bars represent *SD* [Color figure can be viewed at wileyonlinelibrary.com]

Zebrafish Hexb shares ~64% sequence identity with the human protein, and is encoded by a single *hexb* gene. We targeted *hexb* by injecting fertilized zebrafish oocytes at the one‐cell stage with Cas9 protein/gRNA complex targeting exon 1 of *hexb*. To determine the efficiency of targeting the zebrafish *hexb* locus, the generation of insertions and deletions (InDels) was determined by Sanger sequencing genomic DNA from individual 4 dpf larvae (*n* = 24). This was followed by sequence decomposition using TIDE showing that, on average, 94% of the alleles harbor InDels (i.e., mutagenesis efficiency) (Figure 1a) (Brinkman, Chen, Amendola, & van Steensel, 2014). We refer to the F0 generation of larvae that are directly injected with Cas9 protein/gRNA complex as crispants. HEXA and HEXB enzyme activity are both affected in SD patients. Therefore, we analyzed β‐Hexosaminidase (Hexa and Hexb) enzyme activity in dissected heads of control and *hexb* crispants. Tail genomic DNA of these larvae showed highly efficient (97–100%) InDel generation, and, consistent with loss of Hexb activity, *hexb* crispants showed >95% detectable reduction in substrate conversion by β‐Hexosaminidase A + B (Figure 1b).

*HEXB* is a microglial signature gene, and is one of the highest expressed lysosomal enzymes in microglia (Artegiani et al., 2017; Gosselin et al., 2017; Keren‐Shaul et al., 2017; Oosterhof et al., 2017). SD neuropathology includes microglial activation, and therefore we hypothesized that Hexb deficiency would affect microglial lysosomes. To visualize lysosomes in vivo we used lysotracker (LT) staining, which labels all acidic organelles including lysosomes, and to visualize microglia we used transgenic zebrafish in which GFP expression is driven by the *mpeg1‐*promotor (Berg et al., 2016; Ellett, Pase, Hayman, Andrianopoulos, & Lieschke, 2011; Shen et al., 2016). At 4 dpf, although there were no major differences in microglial numbers, lysosomal compartments of *hexb* deficient microglia were found to be enlarged compared to those in controls, suggesting that Hexb deficiency results in lysosomal abnormalities in microglia (Figure 1c,d). Unexpectedly, however, we also observed numerous LT+ speckles outside microglia that were more prominently present in *hexb* crispants (Figure 1c; Movie S1). These speckles are most likely lysosomes of another cell type that is not fluorescently labeled in the *mpeg1* reporter line.

Astrocytes have been shown to accumulate gangliosides GM2 and GA2 in postnatal *Hexb* ^−/−^ mice, and showed increased proliferation (Kawashima et al., 2009). In zebrafish, radial glia has both astrocytic and radial glia/neural stem cell properties (reviewed in: [Lyons & Talbot, 2014]). For example, homologs of two mammalian astrocytes genes, *Slc1a2*, encoding the glutamate transporter GLT‐1, and *Gfap*, are expressed in radial glia in zebrafish (*slc1a2b* and *gfap*) (McKeown, Moreno, Hall, Ribera, & Downes, 2012). Similarly, astrocyte endfeet in mammals express tight junction proteins, which in zebrafish are also expressed particularly in radial glia (Corbo, Othman, Gutkin, Alonso Adel, & Fulop, 2012; Marcus & Easter, 1995). We hypothesized that the lysosomal puncta observed outside microglia in *hexb* crispants may reside in radial glia (RG). We used bacterial artificial chromosome (BAC)‐recombineering to generate an *slc1a2b* fluorescent reporter line, TgBAC(*slc1a2b:Citrine*), expressing Citrine in the cytoplasm of RG (Figure 1d and Figure S1) (Bussmann & Schulte‐Merker, 2011). In *slc1a2b:Citrine* zebrafish, the fluorescent expression pattern appeared highly similar to that observed by *slc1a2b* mRNA in situ hybridization, showing cell bodies lining the ventricle and radial fibers extending throughout the brain (Figure 1d and Figure S1) (Franceschi et al., 2018; Gesemann, Lesslauer, Maurer, Schonthaler, & Neuhauss, 2010; McKeown et al., 2012; Niklaus et al., 2017; Rohrschneider, Elsen, & Prince, 2007). We next visualized lysosomes in RG of control and *hexb* crispants. The majority, >80%, of LT+ speckles, both in control and in *hexb* crispant larvae, showed colocalization with the radial fibers of *slc1a2b* + RG (Movie S2; Figure S2). Quantification of the lysosomal area in the imaged regions, which contain RG fibers and cell bodies, showed more abundant lysosomes present in *hexb* crispants (Figure 1e). Together, loss of Hexb enzyme activity results in abnormal lysosomal phenotypes in both microglia and RG.

### 2.2 Early abnormal lysosomal phenotypes both in microglia and RG of *hexb* ^−/−^ larvae

**Figure 2:**
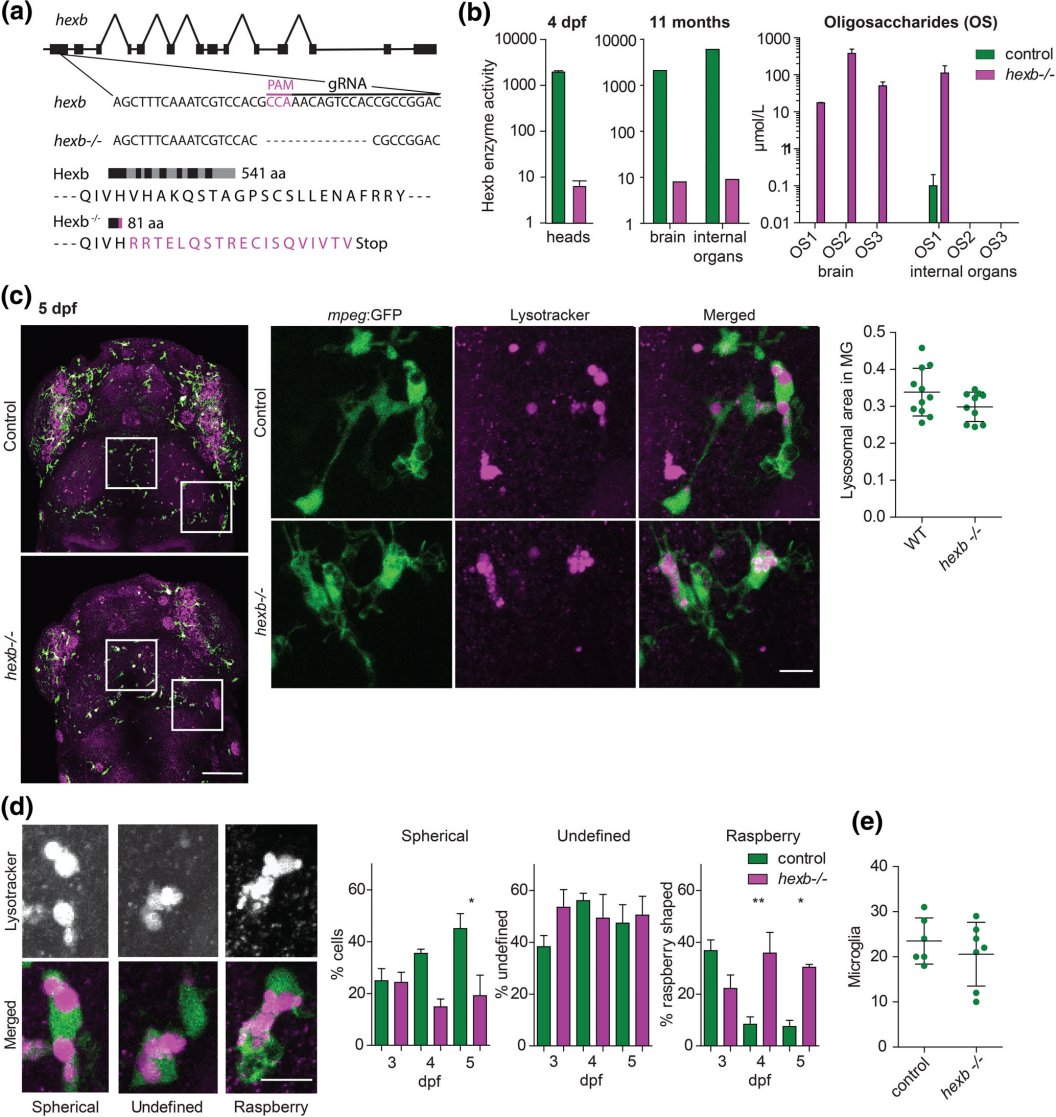
Stable *hexb* ^*−/−*^ fish show impaired Hexb enzyme function and lysosomal abnormalities in microglia. (a) Schematic representation of the *hexb* gene and the 18 bp gRNA and PAM motif. The *hexb* ^−/−^ fish show a 14 bp out of frame deletion disrupting part of the gRNA target sequence and PAM motif, resulting in 19 out of frame amino acids (magenta) followed by a premature stop codon. (b) Enzymatic substrate conversion by β‐Hexosaminidase A + B in larval heads and adult brain and internal organs—containing: intestine, liver, spleen, pancreas and gonads. Mass spectrometry analysis of Hexb deficiency associated oligosaccharide (OS) marker abundance based on exact mass (Hex2‐HexNAc2, 747.267 (OS1); Hex3‐HexNAc3, 1,112.399 (OS2); Hex3‐HexNAc4, 1,315.479 (OS3)) in 1.5 year old *hexb* ^*−/−*^ and control brains and internal organs—containing: intestine, liver, spleen, pancreas and gonads. (c) Representative images of microglia in LT‐stained larvae at 5 dpf, scale bar represents 100 μm. Quantifications were performed on detailed images of depicted regions, scale bar represents 10 μm. Quantification of relative lysotracker area within microglia. (d) Classification of lysosomal morphology in microglia with their quantification at 3, 4, and 5 dpf. *n* = 4 to 6 larvae per group. Scale bars represent 10 μm. (e) Quantification of total microglia in the imaged regions. Error bars represent *SD*. Each dot represents 1 larva [Color figure can be viewed at wileyonlinelibrary.com]

To validate and further investigate the Hexb deficient phenotype, which we observed in *hexb* crispants, we generated stable mutants containing a 14 bp frame‐shifting deletion in the first exon of *hexb*, leading to 19 out of frame amino acids followed by a premature stop codon (Figure 2a). To test whether this causes β‐Hexosaminidase (Hexa + Hexb) deficiency, we measured β‐Hexosaminidase A + B activity in lysates from control and *hexb* ^*−/−*^ larval heads, and also in adult brain and internal organs—containing: intestine, liver, spleen, pancreas, and gonads—, which showed that activity was reduced by >99% (Figure 2b). Consistently, analysis of specific oligosaccharides, which accumulate, as a consequence of HEXB deficiency, in adult brain and internal organs—containing: intestine, liver, spleen, pancreas, and gonads—, showed accumulation in both brain and internal organs from *hexb* ^*−/−*^ fish, but was hardly or not detectable in brain or organs from controls (Figure 2b). Surprisingly, adult *hexb* ^*−/−*^ zebrafish were viable and do not show obvious motility defects.

We wanted to further explore the consequences of Hexb‐deficiency on brain function and development, and determined by LT staining when lysosomal aberrations in microglia first appeared. We first quantified LT‐stained surface in control and *hexb* ^−/−^ microglia in 5 dpf larvae, which in contrast to the small but significant increase in lysosomal volume that we observed in 8 dpf *hexb* crispants, showed no changes (Figure 2c). However, detailed inspection of lysosomes in *hexb* ^*−/−*^ and control microglia showed changes in lysosomal morphology. *Hexb* ^*−/−*^ microglia showed fewer lysosomes consisting of a single round compartment, but more lysosomes that seemed to consist of clustered small lysosomes. To quantify this, we defined three lysosomal categories: spherical (lysosomes that consist of a large, single round compartment), raspberry (lysosomes consisting of clustered smaller compartments) and undefined (lysosomes with intermediate morphology between those categories) (Figure 2d). At 3 dpf, lysosomal morphologies did not differ between controls and *hexb* ^−/−^ brains, with the majority of lysosomes being raspberry shaped (Figure 2d). In control microglia the fraction of raspberry‐shaped lysosomes decreased over time between 3 and 5 dpf, whereas *hexb* ^*−/−*^ microglia contained relatively more raspberry shaped lysosomes, and fewer spherical shaped lysosomes (Figure 2d). This could indicate that small lysosomal vacuoles in *hexb* ^−/−^ microglia fail to fuse and fail to form a larger spherical vacuole, which can possibly be explained by reduced phagocytic flux or reduced autophagosome fusion. Alternatively, lower numbers of microglia could result in increased phagocytic demand for the residual microglia, which could lead to altered lysosomal morphologies. To evaluate this possibility, we quantified microglia numbers. Loss of Hexb activity did not affect microglial numbers (Figure 2e), and therefore lower microglial numbers do likely not cause the changes in lysosomal morphology.

**Figure 3:**
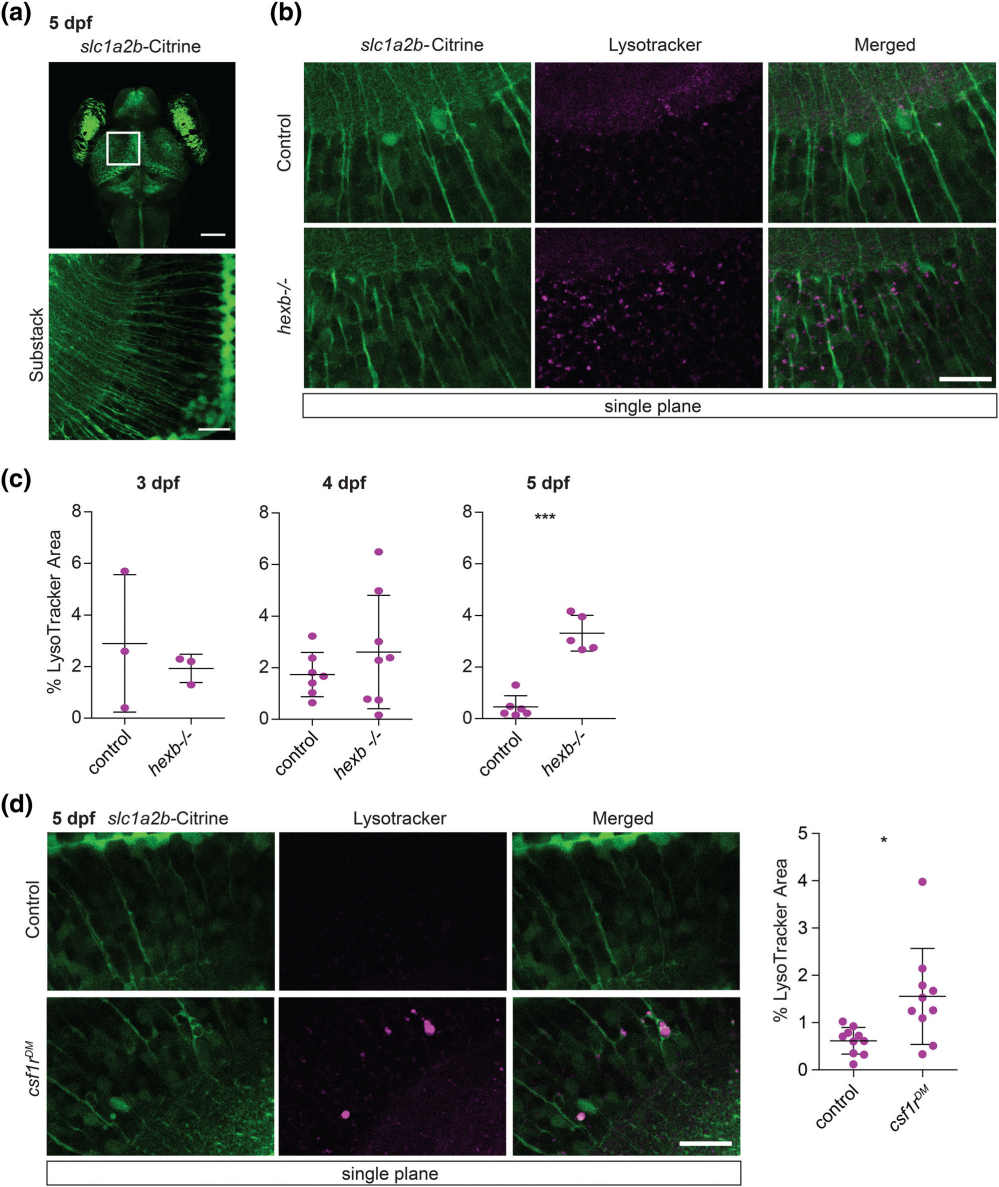
*Hexb* ^−/−^ larvae show increased number of lysosomal speckles in radial glia. (a) Representative image of tgBAC(*slc1a2b:Citrine*) expression in 5 dpf larval brain with magnifications of radial protrusions in the optic tectum. Scale bars represent 100 μm and 20 μm respectively. (b) Representative images of radial glia in LT stained larvae at 5 dpf in areas quantified in (c). Scale bar represents 20 μm. The brightness of the control LT image was enhanced to indicate the presence of some LT+ speckles. (c) Quantification of LT coverage, in percentage, within quantified areas. Each dot represents one larva. Error bars represent *SD*. (d) Representative images of control and *csf1r* ^*DM*^ larvae at 5 dpf, stained with LT and quantification. Scale bar represents 20 μm [Color figure can be viewed at wileyonlinelibrary.com]

To determine when the abnormal lysosomes in RG first appeared, we imaged RG lysosomes in control and *hexb* ^−/−^ zebrafish, and quantified lysosomal volume by measuring LT fluorescence in selected areas (Figure 3a). At 3 dpf LT+ lysosomal speckles were abundantly present in control and *hexb* ^−/−^ radial fibers (Figure 3c). Over time however, LT+ speckles became undetectable in control RG, whereas LT+ speckles in the *hexb* ^*−/−*^ radial fibers became more abundant. At 5 dpf *hexb* ^*−/−*^ RG showed significantly more LT+ speckles than controls did (Figure 3b,c). These LT+ speckles in the radial fibers were highly motile, showing rapid and bidirectional trafficking inside the radial glial fibers, in both controls and *hexb* ^*−/−*^ larvae.

Since the *hexb* ^*−/−*^ larvae presented with lysosomal abnormalities in microglia preceding those in RG, it is possible that abnormalities in RG lysosomes occur secondary to a defect in microglia. Therefore, we analyzed LT‐staining in RG in *csf1r* mutant fish, containing almost no microglia during early development (*csf1r* ^*DM*^) (Oosterhof et al., 2018, 2019). These *csf1r* ^*DM*^ larvae did not retain LT+ speckles in radial fibers of radial glia at 5 dpf, as we did observe in *hexb* ^*−/−*^ larvae (Figure 3b–d). Quantitative analysis however did show increased LT+ staining in RG of *csf1r* ^*DM*^ larvae compared to control larvae (Figure 3d). However, the majority of the LT staining in *csf1r* ^*DM*^ radial glia do not appear as speckles but rather as larger lysosomes (Figure 3d, Movie S5). These lysosomes appear in structures that look like phagolysosomes, suggesting radial glia of *csf1r* ^*DM*^ larvae are phagocytic, but do not retain LT+ speckles in tremendous amounts like we observed in *hexb* mutants (Puñal et al., 2019) (Movie S5, Figure 3b–d). In all, we show that loss of Hexb activity early in embryonic development results in the persistence of enlarged lysosomal speckles in RG fibers, likely independent of microglia.

### 2.3 *hexb* ^−/−^ deficiency causes reduced locomotor activity and increased apoptosis in the brain

**Figure 4:**
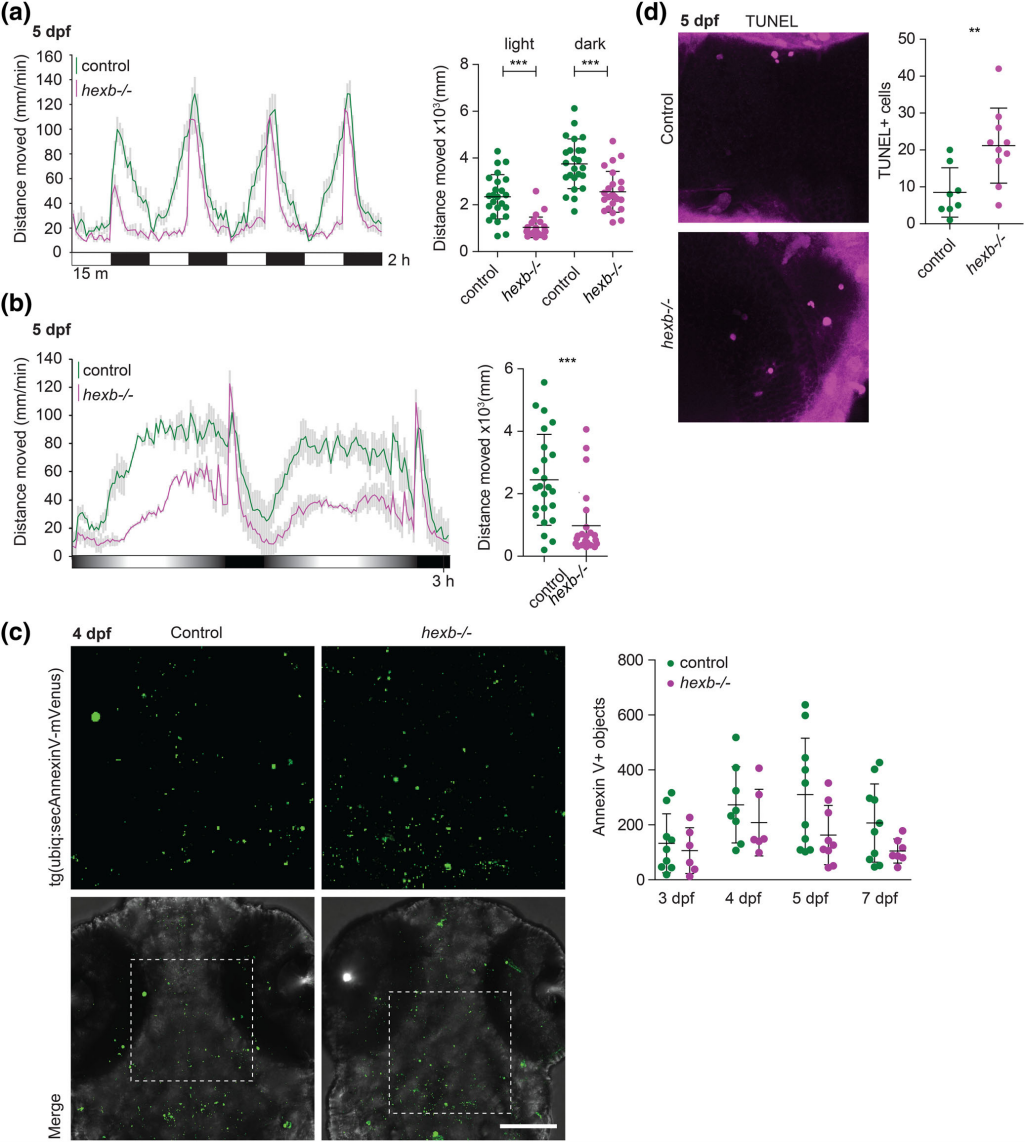
Locomotor activity assay shows abnormal locomotor activity in *hexb* ^−/−^ larvae. (a) Representative graph showing the locomotor responses of larvae to alternating light and dark periods indicated by white and black squares in one single experiment. The dot plot shows the quantification of the sum distance moved during all the light and all the dark periods. (b) Representative graph showing the total distance travelled by larvae during the dusk‐dawn routine (total time: 3 hr 12 min). Quantification of the total distance moved throughout the experiment excluding the dark period. (a, b) *n* = 24 larvae per genotype. Grey shading shows the standard error of the mean. (c) Representative images of control and *hexb* ^−/−^ larvae at 4 dpf transiently expressing secreted annexin V tagged with mVenus. Quantification of annexin V positive objects at 3, 4, 5, and 7 dpf. Scale bar represents 100 μm. (d) Representative images of TUNEL staining at 5 dpf with quantifications. Each dot represents one larva. Scale bar represents 20 μm. Error bars represent *SD* unless stated otherwise [Color figure can be viewed at wileyonlinelibrary.com]

The earliest symptoms that present in SD patients are loss of muscle strength and reduced motor function, both of which are likely caused by atrophy of the innervating lower and/or upper motor neurons. Therefore, we also measured locomotor activity under light and dark conditions in control and *hexb* ^*−/−*^ fish. Zebrafish show stereotypical locomotor responses to shifts in light/dark conditions as early as 24 hpf (Burgess & Granato, 2007; Kokel et al., 2010; MacPhail et al., 2009). Locomotor activity over repeated light–dark cycles was recorded by infrared imaging and measured by automated tracking. Although control and *hexb* ^−/−^ larvae both responded to light/dark switches, *hexb* ^−/−^ larvae showed reduced locomotor activity, which was consistently detected at 4, 5, and 6 dpf (*n* = 24 per group, per experiment) (Figure 4a). This test mainly focuses on the behavioral response to shifts in light intensity, whereas we were mainly interested in general locomotor activity. Therefore, we developed a locomotor assay consisting of gradually increasing and decreasing light intensities, mimicking more physiological relevant changes regulating circadian behavior. *Hexb* ^*−/−*^ larvae showed >50% less locomotor activity during the light periods in this dusk/dawn cycle assay (Figure 4b). These findings indicate that reduced locomotor activity of *hexb* ^*−/−*^ larvae is not due to a defect in the behavioral response to light/dark changes, but due to overall reduced locomotor activity compared to controls. Thus, *hexb* ^*−/−*^ larvae show abnormally low locomotor activity, which coincides with the timing of appearance of glial lysosomal abnormalities in the brain.

SD pathology is characterized by extensive loss of neurons. During normal vertebrate brain development extensive apoptosis occurs, and in zebrafish developmental apoptosis in brain development is particularly high at 3 and 4 dpf (Cole & Ross, 2001; Sierra et al., 2010; Southwell et al., 2012; van Ham, Kokel, & Peterson, 2012; Xu, Wang, Wu, Jin, & Wen, 2016). To determine whether there is more apoptosis in *hexb* ^*−/−*^ brains, we used transgenic zebrafish expressing secreted fluorescently labeled Annexin V to analyze apoptotic cells (Morsch et al., 2015). We did not detect differences in the number of Annexin V+ apoptotic cells or debris at 3, 4, 5, and 7 dpf (Figure 4d). We also performed TUNEL labeling, which stains late‐apoptotic cells, in 5 day‐old brains and observed despite the low numbers of late‐apoptotic cells an ~twofold increase in the *hexb* ^*−/−*^ brains (Figure 4c). Taken together, Hexb deficiency in zebrafish leads to reduced locomotor activity, more late‐apoptotic cell numbers in the brain at Day 5, but no increase in overall apoptotic debris between Days 3 and 7.

## 3 DISCUSSION

Here we generated *hexb* ^*−/−*^ zebrafish, which are β‐Hexosaminidase enzyme‐deficient, and their pathology mimics molecular, cellular and behavioral phenotypes that are also observed in *HEXB‐*deficient Sandhoff disease patients. Already within 5 days of development, *hexb* ^*−/−*^ larvae presented with abnormal lysosomes in microglia and in radial glia. The appearance of abnormal lysosomes in glia coincided with reduced locomotor activity. These phenotypes were observed both in Cas9/*hexb* gRNA injected and in *hexb* ^*−/−*^ larvae, which indicates that crispants are a very suitable method to rapidly screen for pathologic phenotypes, also for other lysosomal or metabolic diseases. Based on our zebrafish model, the early presentation of abnormal lysosomes in glial cells implies that pathogenesis might be characterized by dysfunctional glial cells in the first stages of SD.

Both in controls and *hexb* ^−/−^ RG, we observed abundant lysosomal speckles in the radial fibers, at 3 and 4 dpf. In controls, the lysosomal speckles were largely undetectable by 5 dpf, whereas in *hexb* ^−/−^ fish these abundant RG lysosomes persisted or remained enlarged. Enlarged lysosomal compartments are a characteristic of LSDs. This suggests that, to resolve lysosomes in RG, β‐Hexosaminidase enzyme activity is required, and could imply that extensive processing of sphingolipids such as GM2 occurs at this stage of brain development. To determine whether microglial function might be involved in the resolution of LT+ speckles in radial glia we analyzed *csf1r* ^*DM*^ larvae, which are almost devoid of microglia (Oosterhof et al., 2018). The absence of LT+ speckles in radial glia of microglia‐less mutants shows that the resolution of speckles occurs independently of microglia. Therefore, Hexb activity seems required cell autonomously to resolve LT+ speckles in radial glia. We did however find increased volume of LT staining in RG of *csf1r* ^*DM*^ larvae, but these appear different from the LT+ speckles observed in *hexb* mutants, as they are larger in size and located in phagolysosome‐like structures. Since radial glia or astrocytes may take over phagocytic functions usually performed by microglia, these large LT+ lysosomes could be the result of phagocytic activity of radial glia in the absence of microglia in *csf1r* ^*DM*^ larvae (Puñal et al., 2019). Importantly, RG in vertebrates are neuronal stem cells, and are thought to produce most neurons, but also astrocytes and oligodendrocytes (Doetsch, 2003). This process requires extensive membrane remodeling involving synthesis and degradation of membrane lipids, including glycolipids, in which lysosomes play a key role (Jaishy & Abel, 2016; Kolter & Sandhoff, 2010; Sild, Van Horn, Schohl, Jia, & Ruthazer, 2016). Consistent with this idea, differentiation of RG into astrocytes in mice was accompanied by an increase in lysosomes and autophagosomes (Gressens, Richelme, Kadhim, Gadisseux, & Evrard, 1992). Furthermore, differentiation of SD‐patient iPSC‐derived RG to neurons and astrocytes was skewed towards astrocyte development (Ogawa, Kaizu, et al., 2017). Based on these findings, the persistence or enlargement of lysosomal speckles that we observed in *hexb* ^*−/−*^ RG in vivo, could be the result of impaired glycolipid metabolism, essential for membrane remodeling, and this could influence differentiation towards neurons and glia.

The *hexb* mutant locomotor phenotype is likely the consequence of alterations in the brain, as we did not observe obvious alterations in peripheral motorneurons and neuromuscular junctions (data not shown), whereas aberrant lysosomes in glia are already detected at these early stages. The reduced locomotor activity could be the result of lysosomal dysfunction in either microglia or RG. Microglia have essential functions for brain hemostasis, including clearance of apoptotic cells, myelin remodeling and synaptic pruning (Hagemeyer et al., 2017; Paolicelli et al., 2011; Reemst, Noctor, Lucassen, & Hol, 2016; Safaiyan et al., 2016; Sierra et al., 2010; Wlodarczyk et al., 2017). Defects in such processes could perhaps contribute to defects in locomotor control in the hindbrain leading to locomotor aberrations (Chong & Drapeau, 2007; Kimura et al., 2013). RG on the other hand produce neurons in the brain and alterations in neurogenesis during early development likely affects brain circuitry formation, which could also explain the reduced locomotor activity.

Gangliosidoses due to *HEXA* or *HEXB* mutations are associated with severe neurological symptoms that often precede early lethality. These diseases have been described in several mammalian species, including dogs, cats, and Muntjak deer, and even in birds as Hexa deficiency has been observed in Flamingo's (Cork et al., 1977; Kanae et al., 2007; Kolicheski et al., 2017; Martin et al., 2004; Rahman et al., 2012; Torres et al., 2010; Zeng et al., 2008). *hexb* deficient zebrafish seem to recapitulate early disease stages, but lack extensive neurodegeneration. In addition, adult homozygous *hexb* ^*−/−*^ zebrafish, even though they lack Hexb activity, are viable and do not show obvious behavioral symptoms. To the best of our knowledge, zebrafish are the first vertebrate species tested that do not succumb to pathology caused by loss of Hexb activity. Mice deficient in *Hexa* do not have a severe phenotype as they can bypass the need for HEXBA heterodimer activity by an alternative pathway requiring a sialidase, NEU3, to degrade gangliosides (Seyrantepe et al., 2018). Mass spectrometry analysis of oligosaccharides in zebrafish brains, however, did show the presence of specific oligosaccharides, which accumulate as a consequence of HEXB deficiency, suggesting there is no alternative conversion route active in zebrafish. This suggests that zebrafish are, perhaps to a certain extent, resistant to Hexb deficiency. In addition, zebrafish, in contrast to mammalian species, can regenerate brain tissue and produce new neurons throughout life (Becker & Becker, 2008; Becker & Becker, 2014; Caldwell et al., 2019; Kizil, Kaslin, Kroehne, & Brand, 2012).

In conclusion, *hexb* ^*−/−*^ zebrafish show loss of enzyme activity, accumulation of oligosaccharides, lysosomal abnormalities in glia and reduced locomotor activity, reminiscent of the molecular, cellular, neuropathological, and motility aspects of Sandhoff disease. The very early accumulation of lysosomes in radial processes of RG and raspberry‐shaped lysosomes in microglia, occurring apparently independently of cell death in the brain, may represent early events in pathogenesis. These Hexb deficient zebrafish could be used to further dissect cellular mechanisms that render them resistant to the effects of Hexb deficiency, which leads to disease in various other species, and to identify small molecules that suppress the earliest emergence of cellular pathogenic features.

## 4 MATERIALS AND METHODS

### 4.1 Zebrafish larvae husbandry

Zebrafish embryos and larvae were kept at 28°C on a 14–10‐hour light–dark cycle in 1 M HEPES buffered (pH 7.2) E3 medium (34.8 g NaCl, 1.6 g KCl, 5.8 g CaCl_2_ · 2H_2_O, 9.78 g MgCl_2_ · 6 H_2_O). The medium was changed at 1 dpf to E3 + 0.003% 1‐phenyl 2‐thiourea (PTU) to prevent pigmentation. Larvae used for swimming assays were kept in media without PTU. Transgenic lines used were Tg(*mpeg1*:*GFP*) (Ellett et al., 2011) (Lieschke Laboratory, Clayton, Australia) and TgBAC(*slc1a2b*:*Citrine*)^re01tg^, generated in our lab with an TL background. The *csf1ra* ^*j4e1*/j4e1^ *csf1rb* ^*re01/re01*^ line was used, referred to as *csf1r* ^*DM*^ (Oosterhof et al., 2018; Parichy, Ransom, Paw, Zon, & Johnson, 2000). Animal experiments were approved by the Animal Experimentation Committee at Erasmus MC, Rotterdam.

### 4.2 Generation of the tgBAC(*slc1a2b:Citrine*) reporter line

To generate the tgBAC(*slc1a2b*:Citrine)^re01tg^ reporter line we used the following clone DKEY‐49F8 (HUKGB735F0849Q) in the pIndigoBAC‐536 vector. To perform recombination to insert tol2 sites and the cytoplasmic *Citrine*, the BAC recombineering protocol developed by Bussmann and Schulte‐Merker (2011) was used. Primers are described in Table 1.

**Table 1:** Primers used for BAC recombineering

Primer name	Primer sequence
pIndigoBAC_HA1_iTol2_fw	5′‐ttctctgtttttgtccgtggaatgaacaatggaagtccgagctcatcgctCCCTGCTCGAGCCGGGCCCAAGTG‐3′
pIndigoBAC_HA2_iTol2_rev	5′‐agccccgacacccgccaacacccgctgacgcgaaccccttgcggccgcatATTATGATCCTCTAGATCAGATC‐3′
pTarBAC_HA1_control_fw	5′‐CTGTCAAACATGAGAATTGGTC‐3′
amp_HA1_control_rev	5′‐ACATTTCCCCGAAAAGTGG‐3′
amp_HA2_control_fw	5′‐CTGAGATAGGTGCCTCACTG‐3′
pIndigoBAC_HA2_control_rev	5′‐TGGTGCACTCTCAGTACAATC‐3′
Citrine_HA1_control_rev	5′‐GGACACGCTGAACTTGTGG‐3′
kanR_HA2_control_fw	5′‐TCCTCGTGCTTTACGGTATC‐3′
*slc1a2b* HA1 GFP fw	5′‐GACCCAGATGTGTGCTGTTGTTCATCTGCTCTGTTTCTCCGCAGTTGTGGACCATGGTGAGCAAGGGCGAGGAG‐3′
*slc1a2b* HA2 KanR rev	5′‐TCGATGGGCTCCAGGTGGCTTTCGTGCATCCGTACCTCCACGTGCTTCGGTCAGAAGAACTCGTCAAGAAGGCG‐3′
*slc1a2b* HA1 control fw	5′‐CTCGACTGTGGTGACCTGTG‐3′
*slc1a2b* HA2 control rev	5′‐AGGTTCTTCCGCATCTTCTGG‐3′

### 4.3 CRISPR‐Cas9 genome editing in zebrafish

*Hexb* specific guideRNAs (gRNAs) were designed and synthetized by transcribing from PCR fragment containing a T7 promoter as described previously (Kuil et al., 2018; Moreno‐Mateos et al., 2015). To target the *hexb* gene the online program CRISPRscan was used to design an 18 bp complementary gRNA targeting exon 1 (New Haven, CT) (Moreno‐Mateos et al., 2015) (Table 2). PCR fragments containing a T7 promoter and gRNA sequence were generated by FastStart™ High Fidelity PCR System (Sigma, St. Louis, MO) as described (Moreno‐Mateos et al., 2015). To transcribe gRNAs from PCR fragments the mMESSAGE mMACHINE™ T7 ULTRA Transcription Kit (Invitrogen, Carlsbad, CA) was used according to the manufacturer's instructions followed by RNA cleanup and determination of the quality and concentration by agarose gel electrophoresis and using NanoDrop™ (ThermoFisher, Waltham, MA). *Hexb* specific gRNA 600–900 ng of the synthesized gRNA was mixed with 4 ng of Cas9 nuclease (Sigma, St. Louis, MO). The volume was adjusted with 300 mM KCl to a total volume of 6 μL. Approximately 10 nL of the mix was injected in fertilized zebrafish oocytes. Genomic DNA surrounding the gRNA target site was sequenced using Sanger sequencing and indel efficiency was determined using the online tool TIDE (Brinkman et al., 2014) (Table 2). Founders were selected by sequencing DNA from the tails of adult zebrafish, fish containing mainly 14 bp deletions were selected for breeding. F1 fish were genotyped and selected for −14 bp deletions using sanger sequencing. Mutant fish containing −14 bp mutations in *hexb* are called *hexb* ^*re04*^, but referred to as *hexb* ^*−/−*^ throughout this report.

**Table 2:** Primers used for gRNA synthesis and sanger sequencing

Primer name	Primer sequence
*hexb* gRNA fw	taatacgactcactataGGGTCCGGCGGTGGACTGTTgttttagagctagaa
gRNA synthesis rev	GATCCGCACCGACTCGGTGCCACTTTTTCAAGTTGATAACGGACTAGCCTTATTTTAACTTGCTATTTCTAGCTCTAAAAC
*hexb* sequencing fw	GCTCAACACAACCATGCTCT
*hexb* sequencing rev	ATGTGATCCATACCTGCAGC

### 4.4 LysoTracker staining

Zebrafish larvae were incubated in 1.5 mL tubes with 10 μM LysoTracker™ Red DND‐99 (1:100) (ThermoFisher, Waltham, MA) in 100 μL E3‐PTU. The tube was kept at 28°C for 40 min with the lid open and protected from light. The media was replaced by E3‐PTU only and the fish were mounted after at least 10 min to wash away excess dye.

### 4.5 TUNEL staining

For staining larvae were euthanized on ice, followed by fixation in 4% paraformaldehyde in PBS at 4°C overnight, permeabilized by proteinase K (10 μg/mL) in PBST treatment for 40 min and refixed in PFA for 20 min at room temperature. TUNEL staining was performed as previously described using the Click‐iT TUNEL Alexa Fluor 647 Kit (Invitrogen, Carlsbad) (C10247) (van Ham, Mapes, Kokel, & Peterson, 2010). TUNEL+ cells in the whole brain were quantified.

### 4.6 Plasmid microinjection

To visualize apoptotic cells we injected fertilized oocytes with the ubiq:secAnnexinV‐mVenus plasmid, which was kindly provided by Dr. Marco Morsch, Macquarie University, Sydney (Morsch et al., 2015; van Ham et al., 2010). mVenus positive larvae were selected for confocal microscopy.

### 4.7 Mass spectrometric analysis of oligosaccharides

Tissues were obtained from adult zebrafish, euthanized on ice water, by microdissection as described (Oosterhof et al., 2018). Tissue samples were homogenized in 80 μL water, containing 18 μmol/L tri‐acetylchitotriose and 18 μmol/L hexa‐acetylchitohexaose as internal standards, using a Potter‐Elvehjem tube, followed by sonication at 130 W for 10 s on ice. Insoluble material was removed by centrifugation at 13,000*g* for 10 min. The supernatant was dried under nitrogen flow at 40°C and the resulting residue was dissolved in 80 μL water. The following oligosaccharides were determined semiquantitatively using high‐resolution mass spectrometry: Hex2‐HexNAc2, Hex3‐HexNAc3, and Hex3‐HexNAc4 (Bonesso et al., 2014). Chromatography was performed using a Thermo Scientific Ultimate 3,000 UHPLC system, equipped with a BEH Amide column (Waters 2.1 × 50 mm, 1.7 μm particle size). Analytes were separated by a gradient LC method using mobile phase consisting eluent A (10 mM ammonium acetate in 10:90 water/acetonitrile + 0.1% w/v ammonium hydroxide) and B (10 mM ammonium acetate in 70:30 water/acetonitrile adjusted to pH 9 with ammonium hydroxide) at a flow rate of 0.40 mL/min. The gradient composition was as follows: 1% B at the start, from 1 to 50% B in 1.5 min, from 50 to 95% B in 1 min, 95% B for 2 min, from 95 to 1% B in 0.5 min, and finally 1% B for 4 min before the next sample injection. Injection volume was 0.7 μL and column temperature was maintained at 40°C. Detection was done on a Thermo Scientific QExactive Plus in the full scan mode using the following exact masses (negative mode; ‐H): Hex2‐HexNAc2, 747.267 (OS1); Hex3‐HexNAc3, 1,112.399 (OS2); Hex3‐HexNAc4, 1,315.479 (OS3). For each of these oligosaccharides a single peak was obtained with a mass accuracy <3 ppm. Approximate quantification of oligosaccharides was done by comparison of the oligosaccharide peak area's with that of the internal standards.

### 4.8 Fluorescence microscopy imaging

Zebrafish were anesthetized using tricaine (0.016%) and mounted in 1.8% low melting point agarose. The imaging dish was covered with HEPES‐buffered E3 containing tricaine during imaging. Larvae were imaged on a Leica SP5 intravital microscope with multiphoton laser using a 20× water dipping objective (Leica Plan‐Apochromat, NA = 1.0) using 488 and 561 laser lines. Confocal z‐stack images were acquired. Images were processed with ImageJ software.

### 4.9 β‐Hexosaminidase activity assay

Heads were dissected from 4 dpf control larvae and *hexb* crispants or mutants, after euthanizing them with ice water and a tricaine overdose, and analyzed for enzymatic activity. Adult pools of 2 or 3 dissected heads were grouped according to their genotype and homogenized using sonication in 100 μL H_2_O. For adult organs, animals were euthanized using ice water, Protein concentration was measured using BCA reagent by spectrophotometry after 1‐hr incubation at 37°C (Pierce). The substrate, 5 mM MU‐β‐GlcNAc in 0.2 M Na‐phosphate/0.1 M citrate buffer, pH 4.4 + 0.02% (w/v) NaN_3_, was added to each lysate pool, including one replicate per sample, and incubated for 1‐hr at 37°C in which β‐Hexosaminidase will form 4‐MU. After addition of 0.5 M Na_2_CO_3_ buffer, pH 10.7, 4‐MU fluorescence was measured and normalized to the blank (substrate alone). The final read‐out consisted of nmoles of 4‐MU released per hour per mg of protein.

### 4.10 Locomotor activity assays

To assess the locomotor activity of zebrafish larvae from 3 to 5 dpf, locomotor activity assays were performed using an infrared camera system (DanioVision™ Observation chamber, Noldus) and the using EthoVision® XT software (Noldus). Control (*n* = 24) and *hexb* ^−/−^ (*n* = 24) zebrafish larvae, in 48 well plates, were subjected to two different light/dark routines. The White Light routine consisted of a 30 min habituation period, followed by 4 cycles of 15 min of light (100%)/ 15 min darkness 15 (2.5 hr total). The Dawn routine 30 min habituation in the dark (0% light intensity), followed by the routine described in Table 3 and comprised 3 hr 12 min. Each experiment was performed twice for the three ages (3, 4, and 5 dpf) with *n* = 24 controls, and *n* = 24 mutants per experiment. Distance traveled (mm) per second was measured.

**Table 3:** Dusk‐dawn routine

Light intensity (%)	1	5	10	20	30	40	50	40	30	20	10	5	0
Duration (min)	1	5	5	5	5	5	15	5	5	5	5	5	15

### 4.11 Statistical analysis

To measure *mpeg1*:GFP and LysoTracker volumes in confocal microscopic imaging data, images were analyzed using automatic thresholding in Fiji/ImageJ. The area of the LysoTracker inside *mpeg1*:GFP+ cells was measured, by generating a mask for microglia in FIJI, and compared to the total *mpeg1*:GFP+ area. For the Lysotracker areas of the radial glia, images were automatically thresholded and the total LT+ area per image was counted using the Analyze Particles plug‐in (FIJI). For the radial glia measurements, quantification the total LT+ area relative to total imaged area was used. To quantify the annexin V signal we used the 3D object counter plug‐in (FIJI). For image processing and quantitative analysis, Excel (Microsoft), Prism (Graphpad) and ImageJ were used. Statistical significance was calculated using Student's *t* test. Error bars indicate standard deviation (*SD*) and *p* < .05 was considered significant. Symbols in the graphs (*; **; ***) display levels of significance (*p* < .05; *p* < .01; *p* < .001 respectively).

## CONFLICT OF INTEREST

No authors declare competing interests.

## Supporting information


**Supplementary Figure S1** Representative images of tgBAC(*scl1a2b:Citrine*) expression pattern in larvae of various development stages. Scale bar represents 100 μm

**Supplementary Figure S2** Z‐stack slices showing LT+ puncta (magenta) are inside the radial protrusions of tgBAC(*scl1a2b:Citrine*) + cells (green) depicted by * (3 dpf). Scale bar represents 10 μm

**Supplementary Movie S1** in vivo imaging of lysosomes and *mpeg*‐GFP+ microglia in the optic tectum of a 4 dpf larva showing many LT+ speckles (magenta) outside microglia (green)

**Supplementary Movie S2** Z‐stack showing the majority of LT+ puncta (magenta) are inside the cell bodies or radial protrusions of tgBAC(*scl1a2b:Citrine*) + cells (green) (4 dpf)

**Supplementary Movie S3** 3D visualization of LT+ puncta (magenta) are inside the cell bodies or radial protrusions of tgBAC(*scl1a2b:Citrine*) + cells (green) (4 dpf)

**Supplementary Movie S4** in vivo time‐lapse recordings of 4 dpf control (left) and *hexb^−/−^* (right) microglia showing normal ramified morphology and phagocytic activity (arrows)

**Supplementary Movie S5** Z‐stack showing LT+ staining (magenta) in radial glia (green) of control and *csf1r* ^DM^ 5 dpf larvae showing LT+ puncta (dots) and LT+ lysosomes in phagolysosome‐like structures (arrows)
