# Supplementary figures and images for: Hexb enzyme deficiency leads to lysosomal abnormalities in radial glia and microglia in zebrafish brain development
Source: Glia. 2019 May 29;67(9):1705–18. doi: 10.1002/glia.23641 (PMC6772114; doi:10.1002/glia.23641)

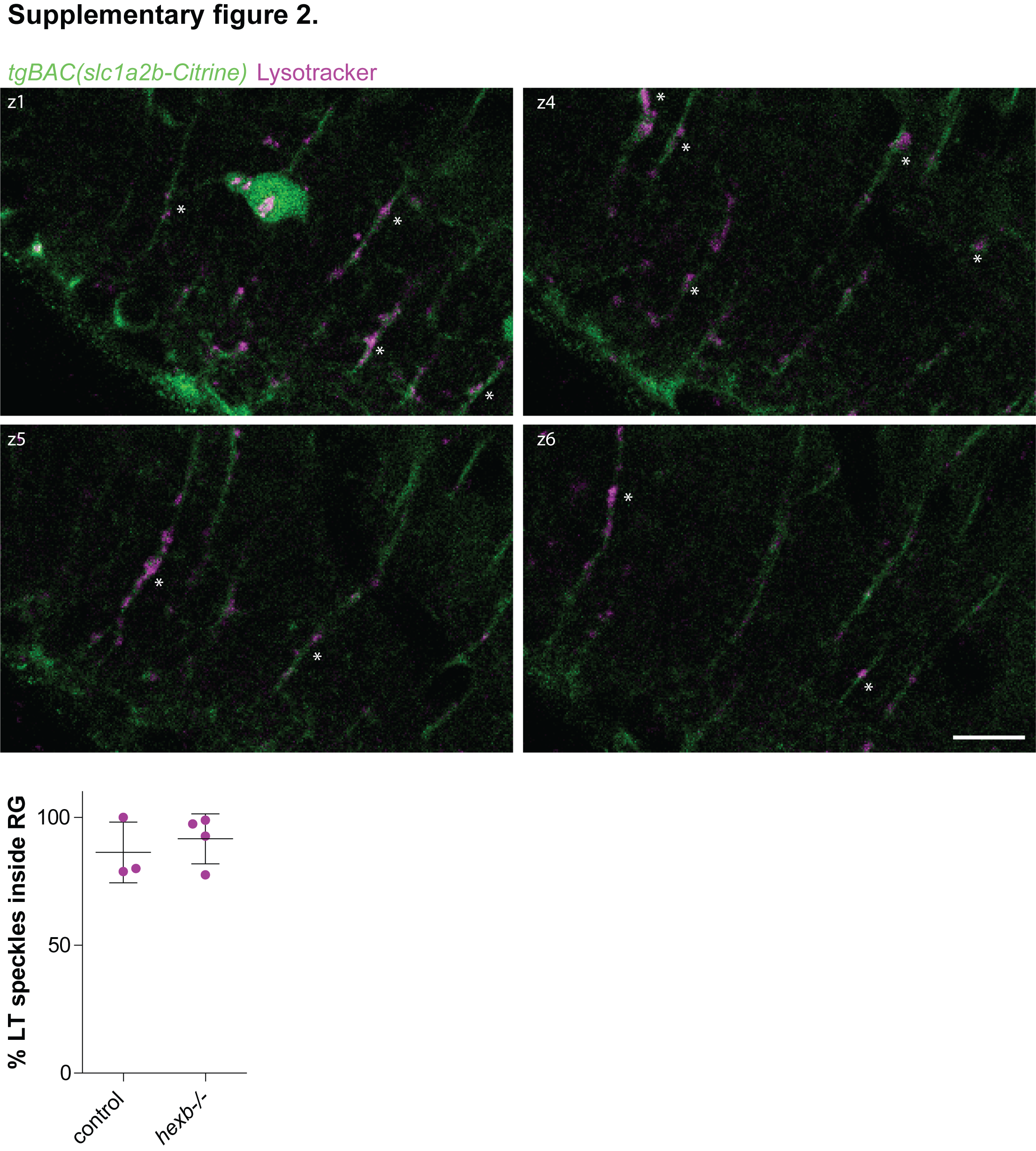

Supplement: Supplementary file 1 — Supplementary Figure S1 Representative images of tgBAC(scl1a2b:Citrine) expression pattern in larvae of various development stages. Scale bar represents 100 μm [file GLIA-67-1705-s001.tif]
